# Redefining and interpreting genomic relationships of metafounders

**DOI:** 10.1186/s12711-024-00891-w

**Published:** 2024-05-02

**Authors:** Andres Legarra, Matias Bermann, Quanshun Mei, Ole F. Christensen

**Affiliations:** 1 CDCB, 4201 Northview Drive, Bowie, MD 20716 USA; 2 Animal and Dairy Science, University of Georgia, 425 River Rd, Athens, GA 30602 USA; 3 Department of Biostatistics, Boston University School of Public Health, Boston, MA 02118 USA; 4 Center for Quantitative Genetics and Genomics, Aarhus University, C. F. Møllers Allé 3, Bld. 1130, 8000 Aarhus C, Denmark

## Abstract

Metafounders are a useful concept to characterize relationships within and across populations, and to help genetic evaluations because they help modelling the means and variances of unknown base population animals. Current definitions of metafounder relationships are sensitive to the choice of reference alleles and have not been compared to their counterparts in population genetics—namely, heterozygosities, *F*_*ST*_ coefficients, and genetic distances. We redefine the relationships across populations with an arbitrary base of a maximum heterozygosity population in Hardy–Weinberg equilibrium. Then, the relationship between or within populations is a cross-product of the form ${\Gamma }_{\left(b,{b}^{\prime}\right)}=\left(\frac{2}{n}\right)\left(2{\mathbf{p}}_{b}-\mathbf{1}\right)\left(2{\mathbf{p}}_{{b}^{\prime}}-\mathbf{1}\right)^{\prime}$ with $\mathbf{p}$ being vectors of allele frequencies at $n$ markers in populations $b$ and $b^{\prime}$. This is simply the genomic relationship of two pseudo-individuals whose genotypes are equal to twice the allele frequencies. We also show that this coding is invariant to the choice of reference alleles. In addition, standard population genetics metrics (inbreeding coefficients of various forms; *F*_*ST*_ differentiation coefficients; segregation variance; and Nei’s genetic distance) can be obtained from elements of matrix ${\varvec{\Gamma}}$.

## Background

Because selection proceeds within breeds, animal breeders have not often dealt with relationship across populations, contrary to evolutionary geneticists, e.g. [1]. Thus, pedigree-based modelling of relationships across animals for genetic evaluation assumed that base populations (Unknown Parent Groups or Genetic Groups) were unrelated and of infinite size. However, populations differ in heterozygosity and are more or less close to each other [2]. In theory, this can be modelled using phylogenetic trees, which can be converted into covariances of gene content at loci [3]. However, these trees are notoriously difficult to estimate in practice. VanRaden [4] proposed methods to model relationships across populations, both within and across breeds, in particular to correctly estimate inbreeding when pedigree information is missing, but his ideas were not broadly applied. With the introduction of genomic evaluation and selection, it was noticed that the assumption of unrelated populations was untenable, and differences across pedigree bases of the different breeds (or groups within breeds) had to be explicitly modelled when pedigree and genomic data were combined. Defining a relationship implies defining a genetic base, which is difficult in practice due to the lack of a clear “starting point”. This motivated the theory of “metafounders” (abbreviated MF in the following) [5–7]. The theory is actually composed of two parts, which are somewhat mixed up in the literature. The first part consists in defining pseudo-individuals (MF) which represent populations. The relationships across these MF, encapsulated in a matrix ${\varvec{\Gamma}}$, model covariances between the means of these populations [6], populations’ homozygosities, and their similarity. These relationships ${\varvec{\Gamma}}$ can be extended via the tabular method [7], in a manner that is a generalization of the regular theory for pedigree relationships, to model covariances across individuals within and across breeds [6, 7], including segregation variances e.g. in F2 animals. The modelling of the covariance across breeds using ${\varvec{\Gamma}}$ implies that the allele substitution effects are defined across breeds [6, 8]. The second part of the theory is the definition of a genetic base from which to define the population means and their covariances. It turns out that a convenient reference is an “absolute” reference point, which is an ideal population with allele frequencies of 0.5 at biallelic markers and therefore with the maximum possible heterozygosity in Hardy–Weinberg equilibrium (HWE) [9]. This is also convenient for compatibility with genomic relationships based on the same 0.5 reference point [6]. The use of 0.5 as a reference leads to a mathematical definition of ${\varvec{\Gamma}}$ as (co)variances of allele frequencies across and within populations [9]. However, this definition is (empirically) sensitive to the choice of reference alleles. In addition, the meaning of ${\varvec{\Gamma}}$ is not yet fully understood in terms of commonly used population genetics metrics, such as inbreeding coefficients, heterozygosity, and genomic relationships across breeds or populations [2]. For instance, a potential user of the theory of MF may be at odds on how to actually compute (or estimate) ${\varvec{\Gamma}}$ from known allele frequencies. Moreover, the user may want to compare inbreeding coefficients or heterozygosities to other population genetics metrics. This is increasingly important with the growing use of genomic measurements for managing genetic diversity [10].

The aim of this short note is to clarify the following two points: (1) give equivalent definitions of ${\varvec{\Gamma}}$ that are invariant to the (maybe non-random) choice of reference alleles; and (2) explain how to interpret ${\varvec{\Gamma}}$ in terms of inbreeding and heterozygosity. These results are used in the companion paper [11] that is dedicated to methods for estimation of ${\varvec{\Gamma}}$ in complex populations.

## Theory

### Definition of ${\varvec{\Gamma}}$ invariant to the choice of reference alleles

The definition of ${\varvec{\Gamma}}$ in [5] can be understood as “the relationship across individuals in the base pedigree population(s), relative to a conceptual base population with all allele frequencies $p=0.5$”. Note that, here, the population for which $p=0.5$ is merely conceptual.

Garcia-Baccino et al. [9] later found out that ${\gamma }_{b,{b}^{\prime}}=8cov\left({p}_{b},{p}_{{b}^{\prime}}\right)$ for populations $b$ and ${b}^{\prime}$. This comes from the fact that the mean and the homozygosity of each population refer to a conceptual base population where the expectation of allele frequencies is $\overline{p} = 0.5$. In other words, some ${p}_{i}$ will be lower than 0.5 and some will be higher, but they average 0.5. This is reasonable to assume, conceptually, by randomly labeling an allele as the reference. However, empirical treatment of observed genomic data often delivers $\overline{p} \ne 0.5$ , even when addressing multiple populations, as populations are real (observed). For this reason, two researchers using different choice of reference alleles for the same dataset may get different numbers from ${\varvec{\Gamma}}$ if they apply blindly ${\gamma }_{b,{b}^{\prime}}=8cov\left({p}_{b},{p}_{{b}^{\prime}}\right)$. The same happens if one uses sequences simulated by coalescence, which call “1” the mutant and “0” the wild allele.

Consider the matrix $\mathbf{M}$ which contains genotypes coded {0,1,2}. The values of genomic relationships obtained as cross-product $\mathbf{G}=\frac{1}{s}\mathbf{Z}{\mathbf{Z}}^{\prime}=\frac{1}{s}\left(\mathbf{M}-{\mathbf{2p}}^{\prime}\right){\left(\mathbf{M}-{\mathbf{2p}}^{\prime}\right)}^{\prime}$ [12] with $s$ a scale factor (typically $s=2\sum {p}_{i}{q}_{i}$ or $s=n/2$ for $n$ markers) are invariant to changes in the reference alleles used to define $\mathbf{M}$ and $\mathbf{p}$. Although rarely explicitly stated, this invariance is well known. We show proof in the Appendix.

In the same spirit, next we need an alternative definition of ${\varvec{\Gamma}}$ which is invariant to the choice of the reference allele. In [7], ${\varvec{\Gamma}}$ and metafounders are defined from alleles in the base-population being sampled from pools of alleles, and counting how many are identical or not. Similarly, for a given labelling of alleles, we need to define unambiguously ${\varvec{\Gamma}}$, without imposing the condition $\overline{p} = 0.5$. To arrive to a meaningful definition, we notice that ${\gamma }_{b,b}$ (the self-relationship of MF $b$) is simply the average (genomic) relationship across animals that form the corresponding base population $b$, and the relationship ${\gamma }_{b,{b}^{\prime}}$ of populations $b$ and ${b}^{\prime}$ is the average relationship across all possible pairs of individuals, one from $b$ and the other one from $b^{\prime}$. This definition was already presented in [13–15] and (unaware of these works) was rediscovered and accommodated to genomic relationships [7].

It follows (as described in the Appendix) that the self-relationship of a population $b$ with itself is ${\gamma }_{b,b}=\frac{1}{s}{\sum }_{k=1}^{n}{\left(2{p}_{b\left(k\right)}-1\right)}^{2} =\frac{1}{s}\left(2{\mathbf{p}}_{b}-\mathbf{1}\right){\left(2{\mathbf{p}}_{b}-\mathbf{1}\right)}^{\prime}$ with $s=\frac{n}{2}$, $n$ being the number of markers, and the relationship across populations $b$ and ${b}^{\prime}$ is ${\gamma }_{b,{b}^{\prime}}=\frac{1}{s}\left({2\mathbf{p}}_{b}-\mathbf{1}\right){\left(2{\mathbf{p}}_{{b}^{\prime}}-\mathbf{1}\right)}^{\prime}$. This is purely a quantitative genetics definition, i.e. ${\varvec{\Gamma}}$ is a feature of the population(s).

Equivalently, we can see ${\varvec{\Gamma}}$ as genomic relationships of the base populations means, seen as individuals, which requires the "genotypes" of each population. If ${\mathbf{p}}_{b}$ is a vector of allele frequencies of the base population $b$, we can see $2{\mathbf{p}}_{b}$ as the “genotype” of the base population. The centered “genotype” of the base population, with respect to the fictitious population with all $p=0.5$, is simply ${\mathbf{z}}_{b}=2{\mathbf{p}}_{b}-\mathbf{1}$ where $1$ is twice 0.5, i.e. the reference allele frequency. Thus, the genomic relationship matrix across populations is simply ${\varvec{\Gamma}}=\frac{1}{s}\mathbf{Z}{\mathbf{Z}}^{\prime}$ where $\mathbf{Z}$ contains twice the allele frequencies of the populations, minus 1: ${z}_{b,k}=2{p}_{b,k}-1$. We note that this is strictly the same definition as in VanRaden [7], if we consider that allele frequencies are “genotypes” of populations—this idea is e.g. in Tier [16]. For statistical inference, ${\varvec{\Gamma}}$ is a parameter of a distribution from which “genotypes” (twice the allele frequencies minus 1) of base populations are sampled.

We also want to stress that if $E\left({p}_{b}\right)=E\left({p}_{{b}^{\prime}}\right)=0.5$, then ${\gamma }_{b,{b}^{\prime}}=\frac{1}{s}\left(2{\mathbf{p}}_{b}-\mathbf{1}\right){\left(2{\mathbf{p}}_{{b}^{\prime}}-\mathbf{1}\right)}^{\prime}=8Cov\left({p}_{b\left(i\right)},{p}_{{b}^\prime\left(i\right)}\right)$ as in [9]. However, the new formulation is more general, and correctly considers the cases where ${\overline{p}}_{b}\ne 0.5$, for instance across several breeds or when one of the “wild” or “mutant” alleles is the reference allele.

### Interpretation of ${\varvec{\Gamma}}$ as heterozygosities or inbreeding coefficients of populations

In this section, we try to relate the values in ${\varvec{\Gamma}}$ to diversity and homozygosity of the population. Consider average heterozygosity of a population, $\overline{\mathcal{H} }=\overline{2{p }_{i}{q}_{i}}$. The conceptual population with $p=0.5$ has ${\overline{\mathcal{H}} }_{max}=0.5$, whereas the observed population $b$ has ${\overline{\mathcal{H}} }_{b}=\overline{\left(2{p}_{b\left(i\right)}{q}_{b\left(i\right)}\right)}.$ We can obtain, after some algebra:

$$\begin{array}{cc}\frac{{\gamma }_{b,b}}{2}=& \frac{1}{2}\frac{2}{n}\sum\limits_{i=1,n}{\left(2{p}_{b\left(i\right)}-1\right)}^{2}=\frac{0.5-\overline{\left(2{p}_{b\left(i\right)}{q}_{b\left(i\right)}\right)}}{0.5}\end{array}=\frac{\left({\overline{\mathcal{H}} }_{max}-{\overline{\mathcal{H}} }_{b}\right)}{{\overline{\mathcal{H}} }_{max}}.$$

From this, it follows that ${\overline{\mathcal{H}} }_{b}={\overline{\mathcal{H}} }_{max}\left(1-\frac{{\gamma }_{b,b}}{2}\right)$, and $\frac{{\gamma }_{b,b}}{2}$ can be seen as an inbreeding coefficient. In other words, $\frac{{\gamma }_{b,b}}{2}$ measures the relative change in heterozygosity from average ${\overline{\mathcal{H}} }_{max}=0.5$ to ${\overline{\mathcal{H}} }_{b}=\frac{1}{2}-\frac{{\gamma }_{b,b}}{4}=\overline{\left(2{p}_{b\left(i\right)}{q}_{b\left(i\right)}\right)}$. Indeed, Jacquard [17] called $\frac{{\gamma }_{b,b}}{2}$ the inbreeding coefficient of a population.

Meuwissen et al. [10] reviewed different measurements of inbreeding for genomic management. Among these, we can find a first inbreeding coefficient based on homozygosity:

$${F}_{hom}=1-\frac{{\mathcal{H}}_{t}}{{\mathcal{H}}_{0}}=1-\frac{1}{n}\sum{\frac{2{p}_{b\left(t,i\right)}{q}_{b\left(t,i\right)}}{2{p}_{b\left(0,i\right)}{q}_{b\left(0,i\right)}}},$$

and when we impose ${p}_{b\left(0,i\right)}={q}_{b\left(0,i\right)}=0.5$, this expression yields:

$${F}_{hom}=1-2\overline{\left(2{p}_{b\left(i\right)}{q}_{b\left(i\right)}\right)}=\frac{{\gamma }_{b,b}}{2}.$$

Thus, $\frac{{\gamma }_{b,b}}{2}$ has the same interpretation as above, i.e. in terms of change in heterozygosity.

The second inbreeding coefficient in [10] is based on drift:

$${F}_{drift}=\frac{1}{n}\sum\limits_{i=1,n}\frac{{\left({p}_{b\left(i\right)}-{p}_{b\left(0,i\right)}\right)}^{2}}{{p}_{b\left(0,i\right)}{q}_{b\left(0,i\right)}},$$

and again, when we impose ${p}_{b\left(0,i\right)}={q}_{b\left(0,i\right)}=0.5$, this yields:

$${F}_{drift}=\frac{1}{n}{\sum }_{i=1,n}\frac{{\left(2{p}_{b\left(i\right)}-1\right)}^{2}}{0.25}=\frac{{\gamma }_{b,b}}{2},$$

identically to the previous one. However, note that here we are imposing ${p}_{b\left(0,i\right)}={q}_{b\left(0,i\right)}=0.5$, which means that, in fact, the value $\frac{{\gamma }_{b,b}}{2}$ is not truly due to genealogical drift from a real, existing population (rather, it describes change from a merely conceptual one), thus describing different values of $\frac{{\gamma }_{b,b}}{2}$ as due to drift would be a misnomer.

The third inbreeding coefficient is defined as follows. If ${\gamma }_{b,b}$ is a relationship coefficient, then:

$${F}_{b}={\gamma }_{b,b}-1,$$

can be seen as an inbreeding coefficient—a measure of homozygosity of the population $b$, not of any individual. Substituting ${\gamma }_{b,b}$ by ${\gamma }_{b,b}=2\frac{0.5-\overline{\left(2{p}_{b\left(i\right)}{q}_{b\left(i\right)}\right)}}{0.5}$ (obtained before) gives:

$$\begin{array}{cc}{F}_{b}=& 1-4\overline{\left(2{p}_{b\left(i\right)}{q}_{b\left(i\right)}\right).}\end{array}$$

If average heterozygosity $\overline{\left(2{p}_{b\left(i\right)}{q}_{b\left(i\right)}\right)}$ is 0, then ${F}_{b}=1$, meaning that there is complete inbreeding and lack of heterozygosity. If average heterozygosity (under HWE conditions) is maximal: $\overline{\left(2{p}_{b\left(i\right)}{q}_{b\left(i\right)}\right)}=0.5$, then inbreeding ${F}_{b}=-1$, meaning complete heterozygosity (under HWE conditions). Again, ${\gamma }_{b,b}-1$ describes a feature of the population—the homozygosity compared to a population in HWE with maximum heterozygosity.

### Interpretation of ${\varvec{\Gamma}}$ in terms of segregation variance, genetic distances and Fst

A commonly used measure of genetic distance across populations is Nei’s minimum genetic distance, ${D}_{b,{b^\prime}}$, which is also the numerator of the ${F}_{ST}$ differentiation index, and is simply [1]:

$${D}_{b,{b^\prime}}=\frac{1}{n}\sum {\left({p}_{b\left(i\right)}-{p}_{b^\prime (i)} \right)}^{2}.$$

After some algebra, we get (as described in the Appendix):

$${D}_{b,{b^\prime}}=\frac{{\gamma }_{b}}{8}+\frac{{\gamma }_{{b^\prime}}}{8}-\frac{{\gamma }_{b{b}^{\prime}}}{4},$$

which also corresponds to the segregation variance, i.e. the difference in genetic variance from F1 to F2 crosses of $b$ and ${b^\prime}$ [7]. Thus, we can use $\gamma$ coefficients to describe genetic distances.

The ${F}_{ST}$ coefficient, applying the Hudson et al. [18] definition as ${F}_{ST}=\frac{\left({H}_{between}-{H}_{within}\right)}{{H}_{between}}$ is shown in the Appendix to be:

$${F}_{ST}=\frac{\frac{{\gamma }_{b}}{8}+\frac{{\gamma }_{b^{\prime}}}{8}-\frac{{\gamma }_{bb^{\prime}}}{4}}{\frac{1}{2}-\frac{{\gamma }_{bb^{\prime}}}{4}},$$

which again shows that $\gamma$ relates to already known descriptors of differentiation. Note that this formula takes into account the covariance of allele frequencies in both populations but also the heterozygosity in each population. For instance, assume two breeds fixed for opposite alleles as follows:

$$\begin{array}{cc}\text{Breed }b& \text{Breed }b^{\prime}\\ A& a\\ c& C\\ D& d\\ e& E\end{array},$$

and so on. We have ${\varGamma }_{b,b}={\varGamma }_{b^{\prime},b^{\prime}}=2$ and ${\varGamma }_{b,b^{\prime}}=-2$. These yield ${F}_{ST}=1$ as expected.

### Other reference base populations

The theory of MF uses 0.5 as the frequency of the reference allele because it is convenient for many purposes. However, one could define relationships from a particular “reference” base population—for instance, in single breed evaluations, it could be the oldest base population in the breed; but it could be a wild ancestor, or an outgroup population. Then, equations should include frequencies in the outgroup (${p}_{o}$) as:

$${\gamma }_{b,b}=\frac{1}{2\sum {p}_{o\left(i\right)}{q}_{0\left(i\right)}}\sum {\left(2{p}_{b(i)}-2{p}_{o(i)}\right)}^{2},$$

$${\gamma }_{b,{b}^{\prime}}=\frac{1}{2\sum {p}_{o\left(i\right)}{q}_{0\left(i\right)}}\sum \left(2{p}_{b(i)}-2{p}_{o(i)}\right)\left(2{p}_{{b}^{\prime}(i)}-2{p}_{o(i)}\right).$$

For MF that describe missing parents across years within breed (typically modelled as unknown parent groups), choosing as reference base population the very first MF in chronological order may be convenient. This would yield a self-relationship of the reference base population of ${\gamma }_{o,o}=0$ and would naturally lead to use the genetic variance of the base population as the parameter of models using ${\varvec{\Gamma}}$ [7]. The problems are (a) ${\varvec{\Gamma}}$ would be no longer full rank and (b) ${p}_{o\left(i\right)}$ is often unknown.

## Discussion

Description of the genetic features of a population in itself is a subject that has not been frequently addressed by animal breeders, because the assumption of unrelated base populations is a simple and efficient one [19], even if the theory could be improved [20–22]. However, the advent of genomic selection led to reconsider modelling means and variances of these populations, in particular because of an acute need for the so-called single step genomic best linear unbiased prediction (ssGBLUP) [23, 24]. At the same time, the concepts of inbreeding, heterozygosity, and drift have been thoroughly revisited with the advent of genomic evaluation [10, 12, 25].

The concept of MF tries to merge the genetic description of populations and the relationships across them [17, 26] with a relationship formulation that can be used for pedigree and genomic selection, giving an explicit modelling to differences in means, segregation variance, or covariances across crossbreds with variable composition. It does this in a manner that is, by construction, compatible (at least in principle) with individual single nucleotide polymorphism (SNP)-based measurements of relationships.

This short note presents an alternative derivation of MF relationships in terms of cross-products of gene content (of the populations), which had not been fully described so far [6, 7, 9]. This derivation is fully compatible to previous derivations and allows to derive estimators more easily for relationships across MF (see in the companion paper [11]). Moreover, we also derive other subproducts that frame our theory with population genetics metrics such as ${F}_{ST}$ or heterozygosity. These relationships have been derived assuming the conceptual base population with $p=0.5$. In addition, the now more coherent theory could be used e.g. to establish priorities for management of diversity across breeds including crosses [27]. Note that whereas values of ${\varvec{\Gamma}}$ itself assume the conceptual base population with $p=0.5$, using them for management of diversity would lead to increase heterozygosities at markers, which may not be desirable [10], whereas on the other hand it gives a unified framework which may be attractive. To solve the issue, Colleau et al. [28] suggested *“…* [converting] the results into more conventional scales…” through scale and shift factors, but that does not resolve the problem of increasing homozygosities versus conserving existing allele frequencies.

On the other side, this theory is somehow compromised because the markers used are not random—they have been tailored, for commercial chips, to be polymorphic in major commercial breeds. For this reason, the relationships obtained in this way, in particular for minor breeds, should not be taken at face value.

Overall, we believe that this note contributes towards a more general and encompassing theory of diversity and relationships, which would be useful both for management diversity and for prediction.

## Conclusions

Metafounders are a concept that describes genetic variation and co-variation within and across finite populations. We presented alternative, new definitions of the concept of MF in terms of cross-product of allele frequencies of populations. The new definitions are more general and can be related to existing concepts of genetic distances, heterozygosity or inbreeding, and they can be naturally integrated into genomic and pedigree-based predictions. We expect that these new definitions will help develop conceptual and practical tools for population management and selection.

## Data Availability

Not applicable.

## Appendix

### Matrix G is invariant to changes in reference alleles

This can be shown as follows. Consider the genotypes of two individuals, row vectors ${\mathbf{z}}_{i}$ and ${\mathbf{z}}_{j}$, which contain values of $-1,0,1$. The genomic relationship of individuals $i,j$ ${G}_{\left(i,j\right)}=\frac{1}{s}{\sum }_{k}{z}_{i,k}{z}_{j,k}$ where $s$ a scaling factor (for instance $s=2\sum {p}_{i}{q}_{i}$; or $s=2\sum {0.5}^{2}=\frac{n}{2}$ with $n$ the number of markers, e.g. assumed to have a frequency of 0.5) and ${z}_{i,k}={m}_{i,k}-2{p}_{k}$ where ${m}_{i,k}=\left\{\mathrm{0,1},2\right\}$ copies of the reference allele and ${p}_{k}$ an assumed frequency for the reference allele at locus $k$. Change of the reference allele results in switching to ${m}_{i,k}^{new}=\left\{\mathrm{2,1},0\right\}$ *i.e*. ${m}_{i,k}^{new}=2-{m}_{i,k}$ and ${p}_{k}^{new}=1-{p}_{k}$. As a result ${z}_{i,k}^{new}={m}_{i,k}^{new}-2{p}_{k}^{new}=-{z}_{i,k}$ and the negative sign cancels at the crossproduct: ${z}_{i,k}^{new}{z}_{j,k}^{new}=\left(-{z}_{i,k}\right)\left(-{z}_{j,k}\right)={z}_{i,k}{z}_{j,k}$. A similar argument holds for the value of $s$, i.e. even if the reference allele is swapped, the values of ${p}_{i}{q}_{i}$ do not change. In particular, the proof does not assume *any* value for allele frequencies. Thus, the value of ${G}_{i,j}$ is invariant to the choice of the reference allele.

### Definition of ${\varvec{\Gamma}}$ invariant to changes in reference alleles

#### Within populations

To define unambiguously ${\varvec{\Gamma}}$ as a function of observed allele frequencies in each base population, without imposing the condition $\overline{p} = 0.5$, we notice that ${\gamma }_{\left(b\right)}$ is simply the average genomic relationship across animals in the corresponding base population $b$. Then, we derive the expected value of the average $\mathbf{G}$ taking into account the allele frequencies in HWE.

First, we consider a single population, $b$. The cross-products ${z}_{i}{z}_{j}$ with scalars ${z}_{i}$ (${z}_{j}$) the genotype at one locus for individual $i$ ($j$) coded as {− 1,0,1} are either 1 (for same homozygotes) or $-1$ (for opposite homozygotes), and these values occur with frequencies that can be obtained from the following Punnet square (here we omit the subindex $b$ for clarity) with crossproducts ${z}_{i}{z}_{j}$ with gamete frequencies of individual $i$ (rows) and $j$ (columns):

$$\begin{array}{cccc}& {p}^{2}& 2pq& {q}^{2}\\ {p}^{2}& 1& 0& -1\\ 2pq& 0& 0& 0\\ {q}^{2}& -1& 0& 1\end{array}.$$

The expected value of ${z}_{i}{z}_{j}$ for the founders of a population is therefore:

$$\begin{array}{cc}{E}_{\text{founders}}\left({z}_{i}{z}_{j}\right)=& {p}^{2}\left({p}^{2}-{q}^{2}\right)-{q}^{2}\left({p}^{2}-{q}^{2}\right)=\\ & \left({p}^{2}-{q}^{2}\right)\left({p}^{2}-{q}^{2}\right)={\left(p-q\right)}^{2}={\left(2p-1\right)}^{2}\end{array}.$$

Then, we sum all $n$ loci and we divide by the scale $s=\frac{n}{2}$ (which is equivalent to assuming a conceptual base population with maximum heterozygosity), which gives:

1 $${\gamma }_{b,b}=\frac{2}{n}{\sum }_{i}{\left(2{p}_{b\left(i\right)}-1\right)}^{2} =\frac{2}{n}\left(2{\mathbf{p}}_{b}-\mathbf{1}\right){\left(2{\mathbf{p}}_{b}-\mathbf{1}\right)}^\prime.$$

for ${\mathbf{p}}_{b}$ the row vector with frequencies in population $b$.

Note that if $\overline{{p}_{b}}=\frac{1}{n}\sum {p}_{b\left(i\right)}=0.5$, this is equivalent (as expected) to $\gamma =8var\left({p}_{i}\right)$ in Garcia-Baccino et al. [9], where random labelling of alleles is assumed, and thus $\overline{{p}_{b}}=0.5$ holds.

Anyway, Eq. (1) is invariant to choosing $p=freq\left(A\right)$ or to choosing $p=freq\left(a\right)$ (in other words, to the choice of reference allele “A” or “a”) since all that it counts is the absolute deviation of ${p}_{b\left(i\right)}$ from $0.5$.

#### Across populations

Now we compute the average genomic relationship across *two* populations in HWE, $b$ and $b^{\prime}$, with respective frequencies ${p}_{b}$ and ${p}_{{b}^\prime}$ as follows:

$$\begin{array}{cccc}& {p}_{{b}^{\prime}}^{2}& 2{p}_{{b}^{\prime}}{q}_{{b}^{\prime}}& {q}_{{b}^{\prime}}^{2}\\ {p}_{b}^{2}& 1& 0& -1\\ 2{p}_{b}{q}_{b}& 0& 0& 0\\ {q}_{b}^{2}& -1& 0& 1\end{array}.$$

This gives that across all founders in both populations:

$$\begin{array}{cc}{E}_{\text{founders}}\left({z}_{i}{z}_{j}\right)& ={p}_{b}^{2}\left({p}_{{b}^\prime}^{2}-{q}_{{b}^\prime}^{2}\right)-{q}_{b}^{2}\left({p}_{{b}^\prime}^{2}-{q}_{{b}^\prime}^{2}\right)\\ & =\left({p}_{b}^{2}-{q}_{b}^{2}\right)\left({p}_{{b}^\prime}^{2}-{q}_{{b}^\prime}^{2}\right)=\left({p}_{b}-{q}_{b}\right)\left({p}_{{b}^\prime}-{q}_{{b}^\prime}\right).\\ & =\left(2{p}_{b}-1\right)\left(2{p}_{{b}^{\prime}}-1\right)\end{array}$$

As before, this is invariant to the reference alleles. For instance, assume that the reference allele is switched, so that the new allele frequency is ${p}^{*}=1-p$. This would give $\left(2{p}_{b}^{*}-1\right)\left(2{p}_{{b}^\prime}^{*}-1\right)=\left(2\left(1-{p}_{b}\right)-1\right)\left(2\left(1-{p}_{{b}^\prime}\right)-1\right)=\left(-2{p}_{b}+1\right)\left(-2{p}_{{b}^\prime}+1\right)=\left(2{p}_{b}-1\right)\left(2{p}_{{b}^\prime}-1\right)$.

Now, summing across all loci and using the scaling $s=\frac{{\text{n}}}{2}$ as before gives:

2 $${\gamma }_{b,{b}^\prime}=\frac{2}{n}\sum \left(2{p}_{b\left(i\right)}-1\right)\left(2{p}_{{b}^\prime \left(i\right)}-1\right) =\frac{2}{n}\left(2{\mathbf{p}}_{b}-\mathbf{1}\right){\left(2{\mathbf{p}}_{{b}^\prime}-\mathbf{1}\right)}^\prime.$$

Again, if $E\left({p}_{b}\right)=E\left({p}_{{b}^\prime}\right)=0.5$, then ${\gamma }_{b,{b}^\prime}=8cov\left({p}_{b\left(i\right)},{p}_{{b}^\prime\left(i\right)}\right)$ as in [9]. However, this formulation is much more general, and correctly considers the cases where ${\overline{p}}_{b}\ne 0.5$, for instance across several breeds or when the “wild” or “mutant” allele is the reference allele.

### Nei’s genetic distance

Nei’s minimum genetic distance, ${D}_{b,b^{\prime}}$, which is also the numerator of the ${F}_{ST}$ differentiation index, is simply:

$${D}_{b,{b}^\prime}=\frac{1}{n}\sum {\left({p}_{b\left(i\right)}-{p}_{b^\prime\left(i\right)} \right)}^{2}.$$

This can be obtained in terms of $\varGamma$ as follows. First, expand the equation above:

$${D}_{b,{b}^\prime}=\frac{1}{n}\sum{\left({p}_{b\left(i\right)}\right)}^{2} +\frac{1}{n}\sum {\left({p}_{{b}^\prime\left(i\right)}\right)}^{2}-\frac{1}{n} 2\sum \left({p}_{b\left(i\right)} - {p}_{b^\prime\left(i\right)}\right).$$

Then express each term as a function of $\varGamma$ coefficients:

$$\frac{1}{n}\sum {\left({p}_{b\left(i\right)}\right)}^{2} =\frac{{\gamma }_{b}}{8}+\frac{1}{n}\sum ({p}_{b\left(i\right)}) -\frac{1}{4},$$

$$\frac{1}{n}\sum {\left({p}_{{b}^\prime\left(i\right)}\right)}^{2}=\frac{{\gamma }_{b^\prime}}{8}+\frac{1}{n}\sum ({p}_{{b}^{\prime}\left(i\right)})-\frac{1}{4},$$

$$\frac{1}{n}\sum \left({p}_{b\left(i\right)} {p}_{{b}^\prime\left(i\right)}\right)=\frac{{\gamma }_{bb^\prime}}{8}+\frac{1}{2}\frac{1}{n}\sum ({p}_{b\left(i\right)})+\frac{1}{2}\frac{1}{n}\sum ({p}_{{b}^{\prime}\left(i\right)})-\frac{1}{4}.$$

Substituting above we obtain:

$${D}_{b,{b}^\prime}=\frac{{\gamma }_{b}}{8}+\frac{{\gamma }_{b^\prime}}{8}-\frac{{\gamma }_{b{b}^\prime}}{4},$$

which corresponds as well to the segregation variance in an F2 from $b$ and ${b}^\prime$ [7].

### Derivation of the ${{\varvec{F}}}_{{\varvec{S}}{\varvec{T}}}$

The ${F}_{ST}$ in Hudson et al. [18] as described by Bhatia et al. [29] is:

$${F}_{ST}=\frac{{H}_{between}-{H}_{within}}{{H}_{between}},$$

where ${H}_{between}-{H}_{within}={D}_{b,b^\prime}=\frac{{\gamma }_{b}}{8}+\frac{{\gamma }_{b\prime}}{8}-\frac{{\gamma }_{bb^\prime}}{4}$ as above. Then, using the identity for $\frac{1}{n}\sum \left({p}_{b\left(i\right)} {p}_{{b}^\prime\left(i\right)}\right)$ above we get:

$$\begin{array}{cc}{H}_{between}=& \frac{1}{n}\sum \left({p}_{b\left(i\right)}\left(1-{p}_{b^\prime\left(i\right)}\right)+\left(1-{p}_{b\left(i\right)}\right){p}_{b^\prime\left(i\right)}\right)\\ =& \frac{1}{n}\sum \left({p}_{b\left(i\right)}\right)+\frac{1}{n}\sum \left({p}_{{b^\prime\left(i\right)}}\right)+-2\frac{1}{n}\sum \left({p}_{b\left(i\right)}{p}_{{b^\prime \left(i\right)}}\right).\\ =& \frac{1}{2}-\frac{{\gamma }_{b,b^\prime}}{4}\end{array}$$

Combining both terms gives:

$${F}_{ST}=\frac{\frac{{\gamma }_{b}}{8}+\frac{{\gamma }_{b^\prime}}{8}-\frac{{\gamma }_{bb^\prime}}{4}}{\frac{1}{2}-\frac{{\gamma }_{b,b^\prime}}{4}}=\frac{{\gamma }_{b}+{\gamma }_{b^\prime}-2{\gamma }_{bb^\prime}}{4-2{\gamma }_{bb^\prime}}.$$
